# Supplementary material for: Exposure to a Farm Environment during Pregnancy Increases the Proportion of Arachidonic Acid in the Cord Sera of Offspring
Source: Nutrients. 2019 Jan 22;11(2):238. doi: 10.3390/nu11020238 (PMC6412650; doi:10.3390/nu11020238)
Supplement: Supplementary file 1 [file nutrients-11-00238-s001.pdf]

Table S1. Proportions of fatty acids in the phospholipid fraction of maternal serum from farming and rural non-farming families.

Fatty Acid Proportions in Maternal Serum; Median, (IQR)			
**Fatty Acid**	**Farmers (n = 22)**	**Non-Farmers (n = 31)**	p [a]
18:2 n-6 (LA)	20 (19–22)	21 (19–22)	0.73
20:3 n-6	3.6 (3.3–4.2)	3.5 (3.1–4.1)	0.64
20:4 n-6 (AA)	6.9 (5.8–7.9)	7.2 (6.3–8.4)	0.14
22:4 n-6	0.30 (0.29–0.38)	0.36 (0.30–0.39)	0.15
22:5 n-6	0.19 (0.16–0.28)	0.21 (0.16–0.34)	0.48
n-6 PUFA, sum	32 (30–33)	32 (30–34)	0.39
n-6 LCPUFA, sum	11 (9–12)	11 (10–12)	0.74
20:5 n-3 (EPA)	1.6 (1.2–1.8)	1.4 (0.9–1.7)	0.24
22:5 n-3 (DPA)	0.71 (0.56–0.85)	0.67 (0.45–0.86)	0.66
22:6 n-3 (DHA)	2.7 (1.4–3.6)	2.6 (2.0–3.7)	0.49
n-3 LCPUFA, sum	4.8 (3.5–6.0)	4.7 (3.8 (5.7)	0.97
PUFA, sum	36 (33–38)	36 (35–38)	0.54
LCPUFA, sum	16 (12–18)	15 (14–17)	0.77
18:1 n-7	1.8 (1.6–2.1)	1.6 (1.5–2.0)	0.23
18:1 n-9	11 (10–12)	11 (8–12)	0.10
Monounsaturated fatty acids, sum	13 (12–14)	12 (12–13)	0.06
18:0	14 (13–15)	14 (13–15)	0.46
20:0	0.43 (0.41–0.48)	0.43 (0.40–0.50)	0.21
22:0	0.92 (0.43–1.1)	0.83 (0.50–1.2)	0.83
Saturated fatty acids, sum	15 (14–16)	16 (14–17)	0.21

Data are presented as medians (interquartile range). [a] Mann-Whitney *U* test. Abbreviations: LA - linoleic acid; AA - arachidonic acid; ALA - alpha-linolenic acid; EPA -eicosapentaenoic acid; DPA - docosapentaenoic acid; DHA - docosahexaenoic acid; PUFA - polyunsaturated fatty acid. LCPUFA - long chain PUFA.

Table S2. Fatty acids in diet 1 month postpartum.

Maternal Intake of Fatty Acids [a]; Median (IQR)			
	**Farmers (n = 23)**	**Non-Farmers (n = 28)**	p [b]
18:0	9.9 (7.7–11)	8.2 (6.1–11)	0.13
20:0	0.28 (0.25–0.38)	0.28 (0.19–0.39)	0.45
18:2 *n*-6 (LA)	9.4 (6.6–12)	9.1 (6.7–12)	0.84
18:3 *n*-3 (ALA)	1.9 (1.6–2.8)	2.3 (1.4–2.9)	0.84
20:4 *n*-6 (AA)	0.11 (0.06–0.17)	0.12 (0.08–0.14)	0.93
20:5 *n*-3 (EPA)	0.02 (0.00–0.09)	0.01 (0.00–0.06)	0.79
22:6 *n*-3 (DHA)	0.11 (0.03–0.30)	0.08 (0.03–0.16)	0.54
22:5 *n*-3 (DPA)	0.05 (0.02–0.07)	0.04 (0.01–0.06)	0.21

Data are presented as medians (interquartile range). [a] Fatty acids calculated from 24 hour dietary recall + 24 hour dietary record. [b] Mann-Whitney *U* test. Abbreviations: LA - linoleic acid; AA - arachidonic acid; ALA - alpha-linolenic acid; EPA - eicosapentaenoic acid; DPA - docosapentaenoic acid; DHA - docosahexaenoic acid.

Table S3. Fatty acids in breast milk obtained 1 month postpartum.

	Breast Milk		
**Fatty Acid**	**Farmers (*n* = 21)**	**Non-Farmers (*n* = 33)**	***p*** [a]
18:2 *n*-6 (LA)	9.0 (7.6–10)	9.5 (8.7–11)	0.27
20:2 *n*-6	0.23 (0.21–0.28)	0.24 (0.22–0.30)	0.50
20:3 *n*-6	0.37 (0.33–0.46)	0.38 (0.31–0.45)	0.38
20:4 *n*-6 (AA)	0.34 (0.32–0.43)	0.39 (0.32–0.44)	0.64
22:4 *n*-6	0.07 (0.05–0.08)	0.06 (0.06–0.08)	0.38
22:5 *n*-6	0.03 (0.02–0.05)	0.03 (0.02–0.04)	0.09
n-6 PUFA, sum	11 (9–12)	11 (10–12)	*0.72*
n-6 LCPUFA, sum	1.1 (1.0–1.3)	1.2 (0.96–1.3)	*0.74*
18:3 *n*-3 (LNA)	1.3 (1.1–1.5)	1.3 (1.0–1.8)	0.64
20:5 *n*-3 (EPA)	0.1 (0.08–0.16)	0.09 (0.07–0.14)	0.31
22:5 *n*-3 (DPA)	0.14 (0.10–0.19)	0.15 (0.10–0.19)	0.85
22:6 *n*-3 (DHA)	0.23 (0.16–0.46)	0.23 (0.20–0.30)	0.97
n-3 PUFA, sum	1.9 (1.7–2.4)	2.1 (1.7–2.5)	*0.97*
n-3 LCPUFA, sum	0.61 (0.56–0.98)	0.66 (0.50–0.82)	*0.35*
PUFA, sum	12 (11–14)	13 (12–15)	0.31
LCPUFA, sum	1.7 (1.6–2.2)	1.8 (1.5–2.1)	0.56
18:1 *n*-7	2.4 (1.9–2.6)	2.2 (2.0–2.4)	0.48
18:1 *n*-9	36 (34–38)	36 (34–38)	0.85
20:1 *n*-7	0.05 (0.04–0.06)	0.05 (0.04–0.6)	0.63
22:1 *n*-9	0.10 (0.08–0.11)	0.10 (0.09–0.12)	0.36
Monounsaturated fatty acids, sum	39 (36–40)	39 (36–41)	0.85
18:0	7.7 (6.7–9.2)	7.5 (6.2–8.4)	0.32
20:0	0.21 (0.17–0.23)	0.22 (0.20–0.26)	0.13
22:0	0.08 (0.07–0.10)	0.09 (0.08–0.11)	0.08
Saturated fatty acids, sum	8.0 (7.0–9.5)	7.8 (6.4–8.8)	0.41

Data are presented as medians (interquartile range). [a] Mann-Whitney *U* test. Abbreviations: LA - linoleic acid; AA - arachidonic acid; ALA - alpha-linolenic acid; EPA -eicosapentaenoic acid; DPA - docosapentaenoic acid; DHA - docosahexaenoic acid; PUFA - polyunsaturated fatty acid. LCPUFA - long chain PUFA.
